# 

**Published:** 1882-07

**Authors:** 


					﻿Yon II.
JULY, 1882.’
No. 7.
JBH.E
HOMEOPATHIC pHY^lClAJl,
A MONTHLY JOURNAL OF MEDICAL SCIENCE.
“ Magna est veritas, el proevalebit."
E. L LEE, ILZE. ID.
EDITOR.
CONTENTS:
(See inside of Cover.)
PHILADELPHIA:
No. 2109 CHESTNUT STREET.
LONDON.
ALFRED HEATH & CO., Sole Agents for Great Britain,
114 Ebury Street, Eaton Square.
Yearly Subscription, $2.00, invariably in advance.
Entered at the Philadelphia Post-Office, as Second Class Mail Matter/
				

